# Early-Life Intervention Using Fecal Microbiota Combined with Probiotics Promotes Gut Microbiota Maturation, Regulates Immune System Development, and Alleviates Weaning Stress in Piglets

**DOI:** 10.3390/ijms21020503

**Published:** 2020-01-13

**Authors:** Quanhang Xiang, Xiaoyu Wu, Ye Pan, Liu Wang, Chenbin Cui, Yuwei Guo, Lingling Zhu, Jian Peng, Hongkui Wei

**Affiliations:** 1 Department of Animal Nutrition and Feed Science, College of Animal Science and Technology, Huazhong Agricultural University, Wuhan 430070, China; xiangquanhang@webmail.hzau.edu.cn (Q.X.); 13586414898@163.com (X.W.); panye19910504@hotmail.com (Y.P.); wangl01@sbtjt.com (L.W.); cuichenbin@webmail.hzau.edu.cn (C.C.); gywmizu@163.com (Y.G.); hul66052668@163.com (L.Z.); 2 The Cooperative Innovation Center for Sustainable Pig Production, Wuhan 400700, China; 3 Hubei Agricultural Sciences and Technology Innovation Center, Wuhan 430070, China

**Keywords:** early-life intervention, fecal microbiota transplantation, gut microbiota, immune system, weaning stress, piglet

## Abstract

Previous studies have suggested that immune system development and weaning stress are closely related to the maturation of gut microbiota. The early-life period is a “window of opportunity” for microbial colonization, which potentially has a critical impact on the development of the immune system. Fecal microbiota transplantation (FMT) and probiotics are often used to regulate gut microbial colonization. This study aims to test whether early intervention with FMT using fecal microbiota from gestation sows combined with *Clostridium butyricum* and *Saccharomyces boulardii* (FMT-CS) administration could promote the maturation of gut microbiota and development of immune system in piglets. Piglets were assigned to control (*n* = 84) and FMT-CS treatment (*n* = 106), which were treated with placebo and bacterial suspension during the first three days after birth, respectively. By 16S rRNA gene sequencing, we found that FMT-CS increased the α-diversity and reduced the unweighted UniFrac distances of the OTU community. Besides, FMT-CS increased the relative abundance of beneficial bacteria, while decreasing that of opportunistic pathogens. FMT-CS also enhanced the relative abundance of genes related to cofactors and vitamin, energy, and amino acid metabolisms during the early-life period. ELISA analysis revealed that FMT-CS gave rise to the plasma concentrations of IL-23, IL-17, and IL-22, as well as the plasma levels of anti-M.hyo and anti-PCV2 antibodies. Furthermore, the FMT-CS-treated piglets showed decreases in inflammation levels and oxidative stress injury, and improvement of intestinal barrier function after weaning as well. Taken together, our results suggest that early-life intervention with FMT-CS could promote the development of innate and adaptive immune system and vaccine efficacy, and subsequently alleviate weaning stress through promoting the maturation of gut microbiota in piglets.

## 1. Introduction

The immune system of newborn animals is generally considered to be immature, which is closely related to a series of early diseases [1,2,3,4]. For instance, the immaturity of the intestinal immune system is regarded as one of the most important reasons for early-life diarrhea and early-weaning stress in mammals [5], and it has been confirmed that the development and maturation of the mammal intestinal immune system occur in infancy, even in the fetal period [6,7,8]. Hence, promoting the maturation of the intestinal immune system in the early-life period is of great significance for the improvement of growth, development, and disease resistance of neonatal mammals.

Gut microbiota plays a vital role in the development and maturation of the intestinal immune system [9,10,11]. Previous research has indicated that colonization history and donor-recipient compatibility play important roles in microbial colonization and community of neonatal mammals [12,13,14]. There has been increasing evidence suggesting that early intervention of the gut microbiota during the critical “window of opportunity” period may be a promising method to improve intestinal microbial colonization [15,16,17,18]. Newborn piglets are usually used as an ideal animal model for studying human nutrition and physiology [19]. In our previous study, early intervention with mature maternal fecal microbiota transplantation (FMT) was found to have positive effects on the improvement of growth performance and immunity and decrease diarrhea in the newborn piglet model [20]. However, the developmental changes in the intestinal microbiota after early intervention remain elusive.

Moreover, aerobic and facultative anaerobic bacteria contribute to the consumption of oxygen in the intestine, which can promote the colonization of strict anaerobes [21,22]. *Saccharomyces boulardii* (*S. boulardii*) is an aerobic yeast [23], and early intervention with *S. boulardii* can alleviate porcine neonatal diarrhea [24,25]. *Clostridium butyricum* (*C. butyricum*) is a probiotic and has been used for the clinical treatment of premature infants with diarrhea by promoting the maturation of the immune function [26,27,28].

Therefore, we hypothesize that the modulation of gut microbiota by mature maternal FMT combined with *C. butyricum* and *S. boulardii* (FMT-CS) oral administration during the early-life period would improve host immune system development and alleviate weaning stress. By using a newborn piglet model, this study might provide novel insights into the improvement of the immune system development in neonatal mammals.

## 2. Results

### 2.1. Bacterial Alpha-Diversity and Beta-Diversity

**Figure 1:**
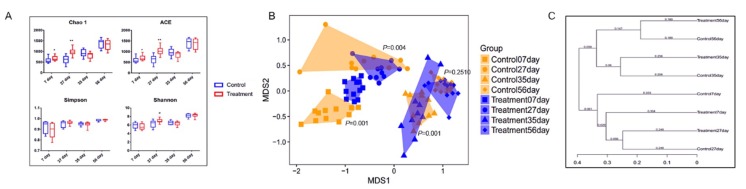
α- and β-diversity of fecal microbiota in piglets after early intervention with FMT-CS. (**A**) Chao1 estimator, ACE estimator, Simpson index, and Shannon diversity index between control group and treatment group. (**B**) NMDS analysis of the fecal microbiota structure between the control groups and treatment groups. (**C**) β-diversity based on the unweighted UniFrac distances of the OTU community.

The fecal microbiota of piglets in the control and treatment groups at the age of 7, 27, 35, and 56 d was analyzed by sequencing the bacterial 16S rRNA V3–V4 region. Early-life intervention with FMT-CS significantly increased the Chao1 estimator and ACE estimator of fecal microbiota at the age of 7 d (*p* < 0.05) and 27 d (*p* < 0.01) in comparison with those in control piglets, and showed a trend to increase the Shannon diversity index at the age of 27 d (0.05 < *p* < 0.1). There was no significant difference in the Simpson index (Figure 1A). The NMDS plot of the dissimilarity of microbial community also revealed a distinct microbial community structure between the control and treatment groups (Figure 1B), and the ANOSIM for differences between the two groups was significant at the age of 7 d (R = 0.7868, *p* = 0.001), 27 d (R = 0.2044, *p* = 0.004), and 35 d (R = 0.3578, *p* = 0.001). However, there was no significant difference in microbial community at the age of 56 d (ANOSIM: R = 0.0283, *p* = 0.251). As shown in Figure 1C, the control piglets showed a higher variability of the OTU community than the treated piglets. Moreover, the fecal microbial community structure at the age of 7 d was more similar to that at the age of 56 d in the treated piglets than in the control piglets.

### 2.2. Early-Life Intervention with FMT-CS Affected the Composition of Fecal Microbiota

**Figure 2:**
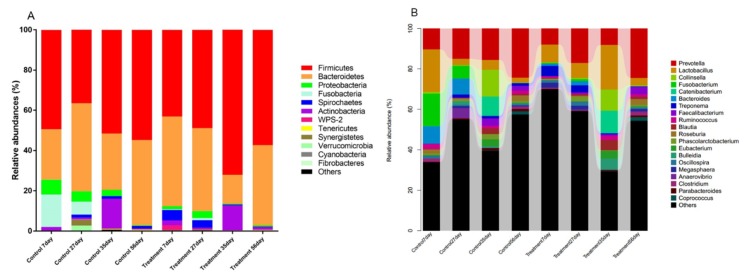
Microbiota composition determined by 16S rRNA gene sequencing of fecal samples. The relative abundance of the phyla (**A**) and genera (**B**) of fecal bacteria present in suckling piglets of both the control and treatment groups.

The microbiota composition of the fecal samples of piglets at the age of 7 d, 27 d, 35 d, and 56 d were assessed by deep sequencing of the V3–V4 region of the 16S rRNA genes. The relative abundance of the fecal microbiota at the phylum and genus levels is displayed in Figure 2. At the phylum level (Figure 2A), *Firmicutes* and *Bacteroidetes* were the most dominant phyla in both control and treated piglets. In treated piglets, the third most dominant phylum was *Spirochaetes* before weaning, and was *Actinobacteria* after weaning. Notably, a rapid increase in the relative abundance of *Actinobacteria* might be a signal of weaning stress. In control piglets, *Fusobacteria* and *Proteobacteria* were the third and fourth most dominant phyla before weaning (7 d and 27 d); while after weaning (35 d and 56 d), the relative abundance of *Fusobacteria* drastically decreased, and *Actinobacteria* became the third most dominant phylum. At the genus level (Figure 2B), *Prevotella* and *Lactobacillus* were dominant in both control and treated piglets. In control piglets, the other two major genera were *Fusobacterium* and *Bacteroides* at the age of 7 d and 27 d, *Collinsella* and *Catenibacterium* at the age of 35 d, and *Roseburia* and *Faecalibacterium* at the age of 56 d; while in treated piglets, the other two major genera were *Treponema* and *p-75-a5*, *Treponema* and *Phascolarctobacterium*, *Collinsella* and *Bulleidia*, and *Faecalibacterium* and *Roseburia* at the age of 7 d, 27 d, 35 d, and 56 d, respectively.

### 2.3. Early-Life Intervention with FMT-CS Affected the Organism-Level Microbiome Phenotypes

**Figure 3:**
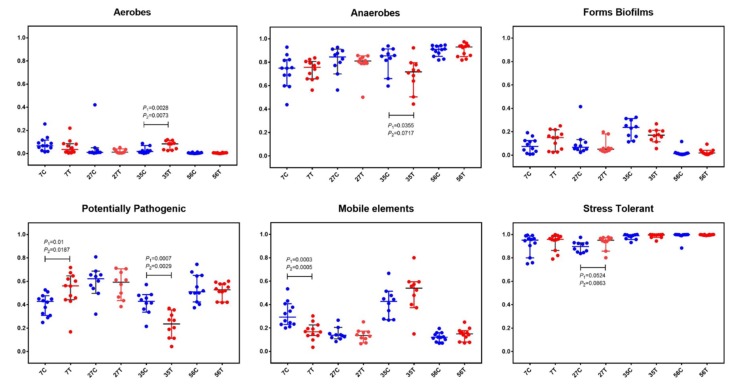
BugBase analysis based on the 16S rRNA gene sequencing dataset. The outcome was grouped according the modules (*x*-axis). Blue dots represent the control group and red dots represent the treatment group. The relative abundance is presented on the *y*-axis. Pairwise Mann–Whitney–Wilcoxon tests (*p*_1_) and FDR-corrected pairwise tests (*p*_2_) were performed for data analysis.

In order to further understand the structure of gut microbiota, BugBase analysis, for analyzing the phenotype of microbiome samples, was performed to predict the proportions of biofilm forming, pathogenic, mobile element containing, oxygen utilizing, and oxidative stress tolerant microorganisms (Figure 3). In both the control and treated piglets, the relative abundance of aerobes gradually decreased while that of anaerobes increased with time. There was a slight decrease of aerobes in treated piglets at the age of 7 d compared with in the control piglets (pairwise Mann–Whitney–Wilcoxon test *p*-value (*p*_1_) = 0.1431; FDR-corrected pairwise *p*-value (*p*_2_) = 0.1822). At the age of 35 d, significant increases in aerobes (*p*_1_ = 0.0028, *p*_2_ = 0.0073) and decreases in anaerobes (*p*_1_ = 0.0355, *p*_2_ = 0.0717) were observed in treated piglets. Notably, significant decreases in potential pathogenic microorganisms (at 35 d; *p*_1_ = 0.0007, *p*_2_ = 0.0029) and mobile element containing microorganisms (at 7 d; *p*_1_ = 0.0003, *p*_2_ = 0.0005) were found in treated piglets.

### 2.4. Early-Life Intervention with FMT-CS Affected the Dominant Bacteria

**Figure 4:**
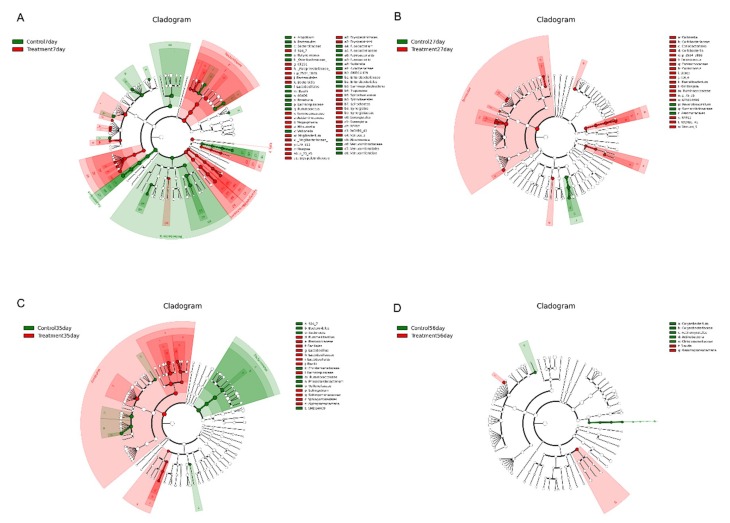
Differential enrichment of fecal microbiota. LefSe analysis of fecal microbiota at the age of 7 d (**A**), 27 d (**B**), 35 d (**C**), and D 56 (**D**) after early intervention with FMT-CS. The cladogram shows the microbial species with significant differences between groups in LEfSe analysis. Red indicates the treatment group and green indicates the control group.

Linear discriminant analysis (LDA) effect size (LefSe) analysis was performed to confirm the effects of early-life intervention with FMT-CS on the fecal microbiota of piglets at 7 d, 27 d, 35 d, and 56 d (Figure 4). The results revealed that at the age of 7 d, *Fusobacteria, Proteobacteria, Bacilli*, and *Verrucomicrobiae* were enriched in control piglets, while *Bacteroidetes, Synergistetes, Spirochaetes, Erysipelotrichi* and *Verruco-5* were dominant in treated piglets (Figure 4A). At the age of 27 d, *Clostridiales, Coriobacteriales*, and *Lactobacillales* were significantly increased in the treatment group (*p* < 0.05), while a significant increase in *Aeromonadales* was observed in the control group (*p* < 0.05). At the genus level, 10 known genera (*Collonsella, Enterococcus, Blautia*, *Coprococcus*, *Dorea*, *rc4-4, Faecalibacterium*, *Oscillospira*, *Ruminococcaceae*, and *p-75-a5*) were significantly increased at 27 d in treated piglets (*p* < 0.05) (Figure 4B). At the age of 35 d, *Lactobacillales, Bacillales*, and *Sphingomonadales* were significantly increased in the treatment group (*p* < 0.05), while *Bacteroidales* and *Clostridiales* showed dramatic increases in the control group. At the genus level, three genera (*Lactobacillus, Rummeliibacillus*, and *Sphingobium*) were significantly increased in the treatment group and *Phascolarctobacterium* showed a significant increase in the control group (*p* < 0.05) (Figure 4C). At the age of 56 d, *Actinobacteria* and *Christensenellaceae* were enriched in control piglets; while the treated piglets showed enrichment of *Gammaproteobacteria* and *Blautia*. Notably, there were obvious increases in some harmful bacteria (*Fusobacterium, Akkermansia*, and *Enterobacteriacease*) in control piglets (Figure 4D).

### 2.5. Early-Life Intervention with FMT-CS Affected the Changing Pattern of Differential Bacteria

**Figure 5:**
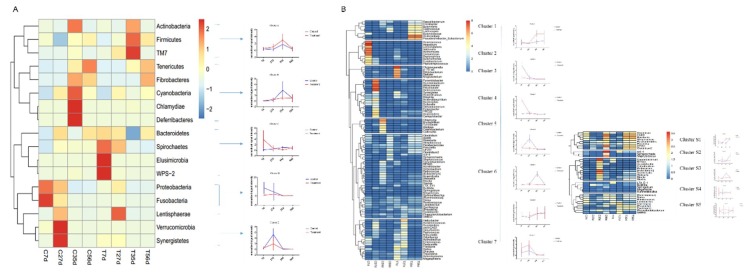
Hierarchical clustering and heatmap of the differential genes at the phylum level (**A**) and genus level (**B**) between groups at different time based on the results of the metastats analysis.

To further analyze the effect of early-life intervention with FMT-CS on the composition of bacteria, we heatmap-analyzed the differential bacteria at the phylum and genus levels based on the results of the metastats analysis (Figure 5), and the relative abundance of differential bacteria is shown in Supplemental Tables S1 and S2. At the phylum level (Figure 5A), five groups of differential bacteria were clustered in the dendrogram. Early-life intervention with FMT-CS increased the relative proportion of cluster A and C, which included more dominant bacteria such as *Firmicutes* and *Bacteroidetes*. In the control group, the relative proportion of harmful bacteria (such as *Fusobacteria* and *Proteobacteria*) was higher, especially in cluster D. At the genus level (Figure 5B), there were seven major clusters, with five sub-clusters for cluster 6. In the treatment group, beneficial bacteria were enriched in the early life of piglets (cluster 3, 7) and post-weaning period (cluster 1). However, the relative proportion of opportunistic pathogens (such as *Fusobacterium* in cluster 2) was high in control piglets. In addition, cluster S1 and S2 contained the two most dominant bacteria, *Prevotella* and *Lactobacillus*, which were enriched in treated piglets at 35 d.

### 2.6. Comparison of Metabolic Pathway Abundance

**Figure 6:**
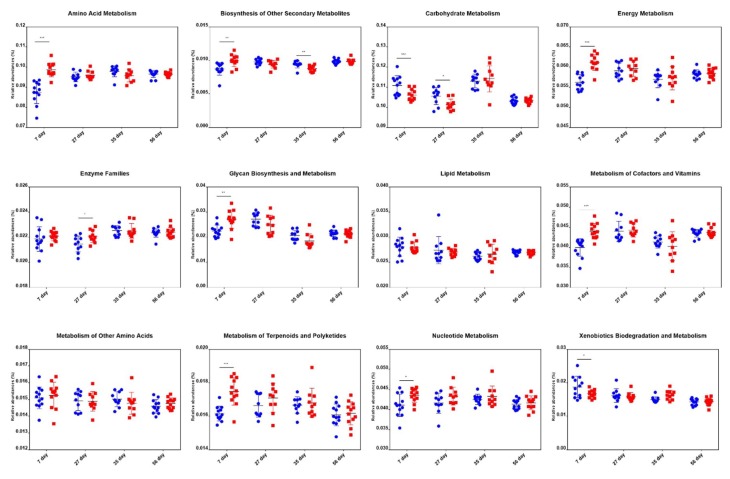
Differences in metabolic functional genes in piglets of the control and treatment groups at the age of 7 d, 27 d, 35 d, and 56 d. Blue dots and red dots represent the control and treatment groups, respectively. * *p* < 0.05, ** *p* < 0.01, *** *p* < 0.001.

We predicted the microbial metagenome with 16S rRNA gene sequencing using phylogenetic investigation of communities by reconstruction of unobserved states (PICRUSt) [29]. The results showed that the genes that regulate the metabolism of vitamins, energy, and glycan, and are highly expressed in adult pigs [30,31], had significantly higher expression in treated piglets. Besides, genes that regulate amino acid metabolism, biosynthesis of other secondary metabolites, metabolism of terpenoids and polyketides, and nucleotide metabolism were more abundant in the treatment group than in the control group at an age of 7 d. However, no significant differences were found for these genes between groups at the age of 27 d, 35 d, and 56 d (Figure 6). These results indicated that the microbiota of the treated piglets was more mature than that of the control piglets.

### 2.7. Early-Life Intervention with FMT-CS Affected Cytokines of the Innate Immune System

**Figure 7:**
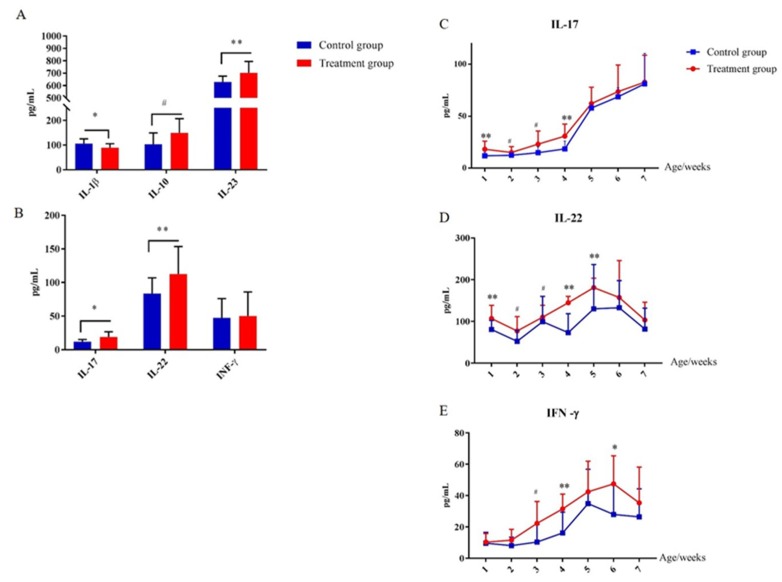
Effect of early-life intervention with FMT-CS on the development of type 3 innate lymphoid cells. (**A**) Cytokines (IL-1β, IL-10, and IL-23) associated with regulation of ILC3, and (**B**) cytokines (IL-17, IL-22, and IFN-γ) secreted by ILC3s in the plasma of piglets at the age of 7 d. (**C**–**E**) Plasma concentrations of IL-17, IL-22, and IFN-γ at indicated time points after birth. Values are expressed as mean ± SEM. ^#^ 0.05 < *p* < 0.1, * *p* < 0.05, ** *p* < 0.01.

In order to evaluate the effect of early-life intervention with FMT-CS on the innate immune system, the plasma cytokines were determined and compared. As shown in Figure 7, at the age of 7 d, plasma IL-23 was significantly increased in treatment piglets compared with in control piglets, while IL-1β was significantly decreased (Figure 7A). In addition, plasma IL-17 and IL-22 were increased in treated piglets as well (Figure 7B). The concentrations of plasma IL-17, IL-22, and INF-γ consistently increased after birth, and reached the maximum at the age of 35 d (Figure 7C–E). Moreover, the concentrations of plasma IL-17, IL-22, and INF-γ of treated piglets were generally higher than those in control piglets.

### 2.8. Early-Life Intervention with FMT-CS Affected the Adaptive Immune Homeostasis

The effects of early gut microbiota intervention on the plasma levels of IgG, IgM, and fecal sIgA are presented in Table 1. Compared with control piglets, the treated piglets showed significant increases in the plasma concentrations of IgG, IgM, and fecal sIgA at 14, 21, 35, and 56 d (*p* < 0.05).

### 2.9. Early-Life Intervention with FMT-CS Affected the Efficacy of Vaccines

**Figure 8:**
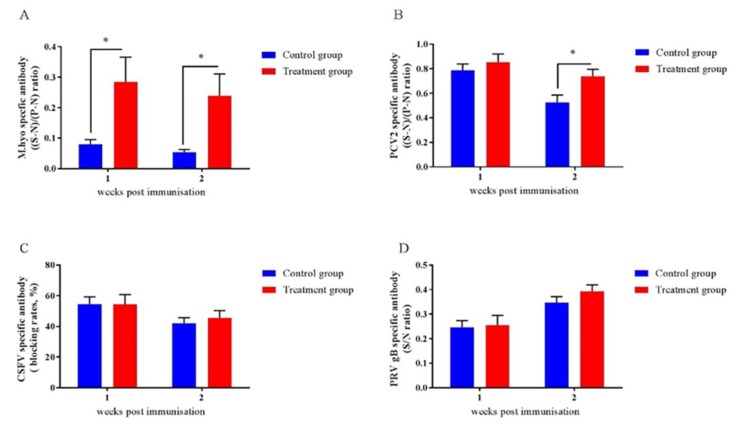
Effects of early-life intervention with FMT-CS on vaccine antibody responses. Commercial ELISA kits were utilized to test anti-M.hyo antibody (**A**), anti-PCV2 antibody (**B**), anti-CSFV antibody (**C**), and anti-PRV antibody (**D**). (S-N)/(P-N) ratio: S, sample OD630; N, negative control OD630; P, positive control OD630. (S-N)/(P-N) _(M.hyo)_ ≥ 0.4, positive; (S-N)/(P-N) _(M.hyo)_ < 0.4, negative. (S-N)/(P-N) _(PCV2)_ ≥ 0.4, positive; (S-N)/(P-N) _(PCV2)_ < 0.4, negative. Blocking rate = (negative control OD450–sample OD450)/negative control OD450. Blocking rate _(CSFV)_ ≥ 40%, positive; blocking rate _(CSFV)_ < 40%, negative. S/N ratio = sample OD450/negative control OD450. S/N _(PRV gB)_ ≤ 0.6, positive; S/N _(PRV gB)_ > 0.6, negative. Values are expressed as mean ± SEM. * *p* < 0.05, ** *p* < 0.01, *** *p* < 0.001.

To assess the impact of early-life intervention with FMT-CS on vaccine responses in piglets, we investigated the antibody responses to four vaccines that are frequently applied to piglets. As shown in Figure 6, the antibodies of M.hyo (Figure 8A) and PCV2 (Figure 8B) were increased in treated piglets, while no differences were detected for anti-CSFV antibody (Figure 8C) and anti-PRV antibody (Figure 8D).

### 2.10. Early-Life Intervention with FMT-CS Affected Weaning Stress

In order to evaluate the effect of FMT-CS on weaning stress, the antioxidant status, intestinal barrier function, and inflammation levels were determined in piglets after weaning (at the age of 35 d). Table 2 shows the effects of early intervention with FMT-CS on CAT, GSH-px, MDA, T-AOC, and T-SOD in the plasma of piglets at the age of 35 d. There were significant increases in CAT (*p* < 0.05), GSH-px (*p* = 0.0514), T-AOC (*p* < 0.05), and T-SOD (*p* < 0.05) levels, and a significant decrease in plasma MDA (*p* < 0.05), suggesting that early intervention with FMT-CS can enhance the antioxidant capacity of piglets after weaning. Besides, FMT-CS significantly reduced the levels of DAO and D-LA in the plasma, indicating that early intervention with FMT-CS may improve the intestinal barrier function of the piglets after weaning. The treatment significantly decreased the levels of TNF-α and IL-6 in the plasma, and enhanced the level of anti-inflammatory factor IL-10. Moreover, a significant decline in plasma cortisol level was detected in the treatment group, indicating that early intervention with FMT-CS may suppress the inflammation in piglets after weaning.

## 3. Discussion

Recently, a number of studies have demonstrated an association between the immune system and gut microbiota [20,32,33,34,35,36,37]. Early infancy is considered as an ideal window for gut microbial colonization [18,38]. Our previous study has validated the benefits of transplanting sow fecal microbiota to newborn piglets [20]. Here, by combining the oral administration of fecal microbiota of gestation sows with *C. butyricum* and *S. boulardii* in newborn piglets, this study for the first time focuses on the promotion of immune development through improving the gut microbiota of piglets. Our results indicated that early intervention with FMT-CS did promote the host immune maturation, and the development of the host immune system was consistent with that of intestinal microbiota.

### 3.1. Early-Life Intervention with FMT-CS Promotes the Maturation of Gut Microbiota

The results showed that early-life intervention with FMT-CS significantly increased the alpha diversity of gut microbiota during the suckling period, representing the positive effect of FMT-CS on the development of gut microbiota during the early life period of piglets [39]. Previous studies have shown that FMT or dietary *C. butyricum* can promote the maturation of intestinal bacteria, which is in accordance with our results [35,40]. In addition, PICRUSt analysis showed that the genes related to the metabolism of vitamins, energy, and amino acids, which represent bacterial maturity [30,31,41], were up-regulated in treated piglets at the age of 7 d. From our results, it can be inferred that early intervention with FMT-CS may accelerate gut microbiota maturation in newborn piglets.

### 3.2. Early-life Intervention with FMT-CS Promotes the Development and Function of Immune System

It has been confirmed that gut microbiota plays a critical role in the development of immune system [18,34], especially the innate immune system [11,42]. We determined several indicators, such as IL-22, IL-17, and IFN-γ, to examine the development of the innate immune system [43,44]. The significant increase in plasma IL-22 and IL-17 may indicate the development of the innate immune system [45,46]. Previous studies have confirmed that the maturation of the intestinal innate immune system of mammals occurs in infancy [7,8,9]. Here, the evaluation of the developmental changes in innate immunity based on the plasma levels of cytokines at different ages revealed that the early intervention with FMT-CS can promote the innate immunity of piglets during the period from birth to weaning.

On the other hand, innate immunity plays an important role in connecting gut microbiota with adaptive immune system development [33,47]. In this study, biomarkers of adaptive immune system were significantly increased in treated piglets. sIgA is produced by gut plasma cells [33]. Gastrointestinal sIgA plays a crucial role in maintaining the intestinal epithelial barrier [48]. It has been shown that fecal sIgA can be quantified as a biomarker of intestinal permeability and immune functions [48]. Besides, early intervention with FMT-CS increased plasma IgG and IgM, and similar results were reported in previous studies of suckling piglets [20,35]. Our results indicate that early intervention with FMT-CS can improve the adaptive immune function of piglets.

Furthermore, it has been indicated that early-life gut microbiota dysbiosis damages vaccine immune responses [49,50]. In this study, we found that early-life intervention with FMT-CS can reduce the risk of gut microbiota dysbiosis, as well as improve the antibody levels of M.hyo and PCV2. The M.hyo and PCV2 vaccines are inoculated during the suckling period of piglets, while the CSFV and PRV vaccines are applied during the post-weaning period, indicating that gut microbiota intervention improves vaccine efficacy mainly in the early-life period. It is worth noting that a significant increase was detected in plasma IgG concentration at the age of 56 d after early intervention. However, there was no significant increase in vaccine efficacy after weaning, which may be due to the fact that IgG and IgM contents are correlated with mucosal humoral immunity [31,51], while specific antibodies are associated with the animals’ tolerance to specific antigens.

In summary, the early intervention of FMT-CS can promote the innate immune development, enhance the acquired immune function, and improve the vaccine efficacy during the suckling period.

### 3.3. Early Intervention with FMT-CS Increases Beneficial Bacteria and Decreases the Opportunistic Pathogens of Piglets

Firmicutes and Bacteroidetes are the core pig microbiome [39,52]. BugBase analysis revealed a slight decrease in aerobes at the age of 7 d, indicating a faster oxygen consumption in the intestines after FMT-CS treatment, which may contribute to the colonization of commensal microflora [21,22]. Similar to the results in previous studies, early intervention with FMT-CS significantly increased the beneficial bacteria and decreased the harmful bacteria [40,53,54,55,56]. The relative abundance of the producers of short-chain fatty acids, such as *Oscillospira*, *Phascolarctobacterium*, *Blautia*, *Coprococcus* and *Faecalibacterium*, was significantly increased, possibly promoting the immune and anti-stress functions [57,58]. Therefore, the piglets may have better ability to cope with the damage caused by weaning stress. *Lactobacillus* plays a key role in disease prevention [59]. After weaning, *Lactobacillus* is the commensal bacteria and is widely used as a probiotic. Fecal *Lactobacillus* is associated with the regulation of the gut immune function and maintenance of the balance of gut microbiota to reduce the inflammatory responses [56,60,61,62,63]. Recent studies have confirmed that FMT could enrich the abundance of gut *Lactobacillus*, which may contribute to the improvement of the intestinal barrier and reduction of diarrhea incidence in piglets [20,35,53]. Moreover, *Lactobacillus* was reported to enhance the gut barrier to confer diarrhea resistance after the weaning of piglets [64]. Therefore, after weaning, significant declines were detected in plasma DAO and D-LA, which are regarded as indicators of intestinal barrier function, suggesting that the piglets in the treatment group developed a stronger intestinal barrier function [65,66,67]. Moreover, the treated piglets showed significant increases in *Lachnospira, Bacillus*, and *Lysinibacillus*, which were found to be associated with the maintenance of gut health and promotion of growth [63,68]. In addition, BugBase analysis showed significant decreases of pathogens in treated piglets, which is consistent with the results of the lefSe and metastats analyses. Potential pathogens, such as *Fusobacteriaceae, Lachnospiraceae, Enterobacteriaceae*, and *Akkermansia*, were significantly decreased in treated piglets, indicating a lower occurrence rate of intestinal dysbiosis [50]. The above results suggest that early-life intervention with FMT-CS increases the relative abundance of beneficial bacteria, while decreases that of opportunistic pathogens.

### 3.4. Early-Life Intervention with FMT-CS Confers Weaning Stress Resistance to Piglets

Previous studies have suggested that weaning can cause oxidative stress-induced damage and inflammation in piglets [69,70,71], which can negatively affect the growth performance of weaned pigs [72]. The gut microbiota plays a vital role in regulating the weaning stress of piglets [64,73]. In order to evaluate to which extent weaning stress may affect the physiological and immune status of piglets, oxidative stress indicators and inflammatory factors were measured. Recent studies have proposed that FMT may be an effective therapeutic approach to eliminate oxidative stress [74]. In this regard, our results also suggest that early intervention with FMT may have a fairly lasting effect on oxidative stress caused by weaning. Moreover, it has been found that FMT can reverse the inflammatory state caused by depression [75]. Besides, early intervention with FMT-CS also tended to increase the plasma level of IL-2, an important survival and proliferation-inducing cytokine for Treg cells [76,77]. In this study, the results demonstrated that early intervention with FMT-CS can suppress the inflammation level in piglets caused by weaning. Overall, it can be concluded that weaning stress can be alleviated by the early intervention with FMT-CS in piglets.

## 4. Materials and Methods

### 4.1. Preparation of Fecal Microbiota Suspension

Six healthy gestation sows (on day 70 of gestation, Landrace × Large white), which had no recent history of gastrointestinal diseases and antibiotics treatment in more than 2 months, were used as fecal donors in this experiment. The fresh fecal of donor sows was collected separately after 12 h of fasting. The fecal suspension was prepared as described by Pang et al. (2007) [78]. In brief, fresh fecal sample was immediately diluted 20-fold in 0.1 M sterile phosphate-buffered saline containing 10% glycerol (PBSG). Then, 1.0 × 10^9^ CFU/mL *C. butyricum* and 1.0 × 10^9^ CFU/mL *S. boulardii* were added into the fecal suspension, which was used as the transplanted bacterial suspension (TBS) and stored at −80 °C.

### 4.2. Animal Experimental Design

The protocol for the animal experimental procedures was approved by Institutional Animal Care and Use Committee of Huazhong Agricultural University (Wuhan, China). The ethical number of this study is HZAUSW-2018-013.

One hundred and ninety healthy neonatal piglets (Duroc × Landrace × Large white) with an average birth weight of 1.55 ± 0.31 kg were obtained from 22 sows with the parity between 2 and 5. All piglets were raised under the same conditions on a commercial farm in Guangxi Province, China. In order to avoid maternal differences, the piglets from 22 litters were randomly divided into two groups: the control group (*n*_sows_ = 10, *n*_piglets_ = 84) and the treatment group (*n*_sows_ = 12, *n*_piglets_ = 106). In the first three days after birth, each piglet in the treatment group was orally administered with TBS daily at the volume of 2 mL, while the piglets in the control group were given the same frequency and volume of PBSG. Piglets were weaned at the age of 28 d. A total of 149 weaned piglets (*n*_control_ = 71, *n*_treatment_ = 78) were randomly raised in eight similar pens, with four pens for each group. The experiment was ended at the age of 56 d. During the experiment period, the piglets were allowed free access to water and antibiotics-free diets.

### 4.3. Immunization

The piglets were immunized with the following inactivated vaccines: mycoplasma hyopneumoniae (M.hyo) vaccine at the age of 8 d, porcine circovirus type 2 (PCV2) vaccine at the age of 15 d, classical swine fever virus (CSFV) vaccine at the age of 42 d, and swine pseudorabies virus (PRV) vaccine at the age of 49 d.

### 4.4. Sample Collection

Blood samples were collected from 12 randomly selected piglets from the control and treatment groups by the vena jugularis into heparin vacuum blood tubes. The samples were then centrifuged at 3000× *g* for 30 min, and the plasma was carefully separated and stored at −80 °C until analysis. Fecal samples were collected at the age of 7 d and 56 d from 12 randomly selected piglets of the control and treatment groups, and stored at −80 °C until analysis.

### 4.5. DNA Extraction, 16S rRNA Gene Amplification, and Illumina MiSeq Sequencing

Microbial DNA was extracted from intestinal contents using a QIAamp DNA Stool Mini Kit (Qiagen, Germany) following the manufacturer’s protocols. Successful DNA extraction was confirmed by 0.8% agarose-gel electrophoresis. The V3–V4 hypervariable region of the bacterial 16S rRNA gene was amplified using primers 341F (5′-ACT CCT ACG GGA GGC AGC AG-3′) and 806R (5′-GGA CTA CHV GGG TWT CTA AT-3′). The PCR conditions were predenaturation at 98 °C for 2 min, 25 cycles of denaturation at 98 °C for 15 s, annealing at 55 °C for 30 s, elongation at 72 °C for 30 s, and a final post-elongation cycle at 72 °C for 5 min. The PCR products were purified with AMPure XP beads (AXYGEN). After purification, the PCR products were used for the construction of libraries and then paired-end sequenced on Illumina Miseq (Illumina, CA, USA) at the Personalbio, Shanghai, China.

### 4.6. Analysis of Sequencing Data

Alpha diversity (including four indices: Chao1, Shannon, Simpson, and ACE) of bacterial richness and diversity was analyzed using QIIME (Caporaso et al., 2010). Beta diversity was analyzed by PCA based on operational taxonomic unit (OTU), and the hierarchical clustering tree was constructed based on unweighted Unifrac distances. Bray–Curtis distances were calculated based on the square root-transformed OTU relative abundance and used to generate the non-metric multidimensional scaling (NMDS) ordination plot. All of these indices in our samples were calculated with QIIME (Version 1.7.0) and displayed with R software (Version 2.15.3). On the basis of classified information, a histogram of linear discriminant analysis (LDA) distribution was implemented by the LDA effect size analysis (LEfSe) to find the bacteria with significant differences in relative abundance between two groups. Microbial phenotypes were predicted with BugBase [79], a software that relies on the tools PICRUSt, IMG, KEGG and PATRIC.

### 4.7. Measurements of Cytokines Related to Innate and Adaptive Immunity Development and Antibody Responses

sIgA, IgM, IgG, IL-22, IL-17, and IFN-γ were measured by ELISA kits (mlbio good elisakit producers, Shanghai, China) following the manufacture’s protocol. Antigen-specific IgG was measured using commercially available ELISA kits including the M.hyo IgG ELISA kit (IDEXX Laboratories, GP521, America), PCV2 IgG ELISA kit (zcwdbio, 180801, Wuhan, China), CSFV IgG ELISA kit (IDEXX Laboratories, M301, America), and PRV IgG ELISA kit (Keqian Biology Co., Ltd., 181010, Wuhan, China) following the manufacture’s protocol. Data were analyzed using GraphPad Prism 7.01 (GraphPad Software Inc., La Jolla, CA, USA). A two-tailed Student’s t test was used to assess the significance.

### 4.8. Intestinal Barrier Function

The levels of plasma diamine oxidase (DAO) and D-lactate (D-LA) were examined using an ELISA test kit (m1002413 and m1647900, mlbio good elisakit producers, Shanghai, China) according to the manufacturer’s instructions. The absorbance was determined with a 96-well microtiter plate reader INFINITE 200 PRO (Tecan Austrla Gmbh, Untersbergstr. 1A, A-2082 Grödlg, Austria).

### 4.9. Antioxidant Indices

Glutathione peroxidase (GSH-px), catalase (CAT), total antioxidant capacity (T-AOC), malondialdehyde (MDA), and total superoxide dismutase (T-SOD) of plasma were examined using an assay kit (Nanjing Jiancheng Bioengineering Institute (Nanjing, China)) according to the manufacturer’s instructions.

### 4.10. Inflammation Levels

The contents of plasma cortisol, pro-inflammatory cytokines (Plasma INF-γ, TNF-α, and IL-6), and anti-inflammatory cytokines (plasma IL-2 and IL-10) were examined using an ELISA test kit (mlbio good elisakit producers, Shanghai, China) according to the manufacturer’s instructions.

### 4.11. Statistical Analysis 

Before the analysis, Kolmogorov–Smirnov and Levene tests were performed for the normality and heteroscedasticity of the data. Cytokine values were assessed by ANOVA. The procedure for the repeated measurements of SAS 9.4 (SAS Institute, Inc., Cary, NC, US) was used. Data were expressed as means ± SEM. Statistical significance was defined as *p* < 0.05, whereas *p* values between 0.05 and 0.10 were considered as a trend.

## 5. Conclusions

Our results reveal that the early-life intervention with FMT-CS regulates gut microbiota structure, accelerates the maturation of gut microbiota, and reduces the risk of gut microbiota dysbiosis in piglets. We also found that FMT-CS can promote the development of the immune system and increase vaccine efficacy during the early-life period of piglets, so as to alleviate the damage of weaning stress. Our findings provide an important reference for the regulation of innate and adaptive immunity development, vaccine efficacy, and weaning stress through early-life gut microbiota intervention in the pig industry. However, further studies are still needed to better understand the mechanism by which gut microbiota regulates the development of innate immune cells.

## Figures and Tables

**Table 1:** Effects of early intervention with FMT-CS on fecal sIgA, plasma IgG, and IgM concentrations of piglets.

Item	Control	Treatment	SEM	*p*-Value
Day 14				
Fecal sIgA, μg/g	128.24 ^b^	168.53 ^a^	1.35	< 0.0001
Plasma IgG, mg/mL	19.8 ^b^	26.94 ^a^	1.47	0.0135
Plasma IgM, mg/mL	15.48 ^b^	22.46 ^a^	1.1	< 0.0001
Day 21				
Fecal sIgA, μg/g	114.19 ^b^	145.92 ^a^	1.8	0.0242
Plasma IgG, mg/mL	20.56 ^b^	26.28 ^a^	1.08	0.0052
Plasma IgM, mg/mL	11.1 ^b^	17.86 ^a^	1.12	0.0004
Day 35				
Fecal sIgA, μg/g	132.09 ^b^	148.78 ^a^	3.25	0.0071
Plasma IgG, mg/mL	13.74 ^b^	24.08 ^a^	1.22	< 0.0001
Plasma IgM, mg/mL	10.95 ^b^	18.03 ^a^	0.95	< 0.0001
Day 56				
Fecal sIgA, μg/g	102.24 ^b^	128.12 ^a^	3.85	0.0001
Plasma IgG, mg/mL	16.92 ^b^	19.05 ^a^	0.41	0.0058
Plasma IgM, mg/mL	9.91 ^b^	13.69 ^a^	0.58	< 0.0001

^a,b^ Values within a row with different superscripts differ significantly at *p* < 0.05, All values are presented as means ± SEM.

**Table 2:** Effect of early-life intervention with FMT-CS on weaning stress of piglets.

Items ^1^	Biomarkers	Control	Treatment	SEM	*p*-Value
Antioxidant Indices	CAT, U/mL	25.14 ^b^	33.22 ^a^	1.81	0.0218
	GSH-px, U/mL	368.38	469.15	26.12	0.0514
	MDA, nmol/mL	7.81 ^a^	5.30 ^b^	0.53	0.0147
	T-AOC, U/mL	36.22 ^b^	48.70 ^a^	2.94	0.0302
	T-SOD, U/mL	14.58 ^b^	35.59 ^a^	1.44	0.0271
Intestinal Barrier Biomarkers	DAO, ng/mL	374.18 ^a^	296.8 ^b^	17.513	0.0115
	D-LA, umol/mL	880.16 ^a^	631.27 ^b^	19.84	0.0004
Inflammation Levels	IFN-γ, pg/mL	70.99	59.43	2.72	0.0530
	TNF-α, pg/mL	443.13 ^a^	245.89 ^b^	9.42	< 0.0001
	IL-6, pg/mL	1152.04 ^a^	900.18 ^b^	34.09	0.0044
	IL-2, pg/mL	610.79	729.64	36.86	0.1640
	IL-10, pg/mL	374.69	493.06	27.96	0.0713
	Cortisol, ng/mL	272.73 ^a^	210.05 ^b^	8.59	0.0022

^1^ All of these biomarkers were determined at the age of 35 d. ^a,b^ Values within a row with different superscripts differ significantly at *p* < 0.05, All values are presented as means ± SEM.

## Supplementary Materials

Supplementary materials can be found at https://www.mdpi.com/1422-0067/21/2/503/s1.

## Abbreviations

FMT	Fecal microbiota transplantation
FMT-CS	FMT combined with *C. butyricum* and *S. boulardii*
GSH-px	Glutathione peroxidase
CAT	Catalase
T-AOC	Total antioxidant capacity
MDA	Malondialdehyde
T-SOD	Total superoxide dismutase
D-LA	D-lactate
DAO	Diamine oxidase
PBSG	Phosphate-buffered saline containing 10% glycerol
TBS	Transplanted bacterial suspension
