# Supplementary material for: Early-Life Intervention Using Fecal Microbiota Combined with Probiotics Promotes Gut Microbiota Maturation, Regulates Immune System Development, and Alleviates Weaning Stress in Piglets
Source: Int J Mol Sci. 2020 Jan 13;21(2):503. doi: 10.3390/ijms21020503 (PMC7014131; doi:10.3390/ijms21020503)
Supplement: Supplementary file 1 [file ijms-21-00503-s001.pdf]

Supplemental table 1 Relative abundance of phylum level, %

Item	Control				Treatment			
	7d	27d	35d	56d	7d	27d	35d	56d
Actinobacteria	1.8066	0.8103	14.758	0.5207	2.3507	0.7908	12.3195	1.3994
Bacteroidetes	25.0282	43.8504	28.0948	42.3201	44.5531	41.2563	14.3936	39.7971
Chlamydiae	0	0	0.6598	0	0	0.0102	0.039	0
Cyanobacteria	0	0.0253	0.0535	0.0173	0	0.0225	0.0101	0.019
Deferribacteres	0	0	0.0061	0	0	0	0	0
Elusimicrobia	0	0	0	0	0.0101	0	0	0
Fibrobacteres	0	0	0.0155	0.0122	0	0	0	0.0147
Firmicutes	49.458	36.4965	51.5011	54.789	43.0871	48.8044	72.0047	57.3556
Fusobacteria	16.135	6.2449	0	0	0.3381	1.0349	0.002	0
Lentisphaerae	0.0017	0.0598	0.0041	0.0009	0.0067	0.089	0	0.0009
Proteobacteria	7.3126	5.1818	0	0.2663	1.5088	3.45	0.3838	0.5752
Spirochaetes	0.0225	1.6235	1.3094	1.3126	5.1963	3.724	0.5303	0.2735
Synergistetes	0.0242	3.0305	0.0267	0	0.4553	0.5469	0.001	0
Tenericutes	0	0.0323	0.3508	0.7291	0.0159	0.0236	0.2782	0.5629
TM7	0	0	0.0041	0	0.0017	0.0092	0.0233	0.0009
Verrucomicrobia	0.2112	2.5999	0.0226	0	0.0529	0.1075	0.0124	0.0009
WPS-2	0	0.0284	0.1595	0	2.4233	0.1287	0	0

Supplemental table 2 Relative abundance of genus level, %

Item	Control				Treatment			
	7d	27d	35d	56d	7d	27d	35d	56d
Actinomyces	0.7882	0	0	0	0.1218	0	0	0
Corynebacterium	0.0226	0.0063	0.3348	0.0353	0.1752	0.013	0.1667	0.0126
Rothia	0.0693	0.0032	0	0	0.1376	0.0456	0	0
Bifidobacterium	0.1392	0.7604	0	0	3.2092	0.2097	0.0028	0
Atopobium	0.1321	0	0	0	0	0	0	0
Collinsella	1.2515	0.6854	20.2592	0.3721	0.6581	1.363	13.2438	0.8291
Slackia	0.0059	0	0.0617	0.0064	0.0124	0.009	0.0769	0.0415
Butyricimonas	1.2764	0.5765	0.0015	0	0.428	0.2325	0.0011	0
Odoribacter	0.0456	0.0581	0	0	0.0748	0.0914	0	0
CF231	0.1161	0.9799	0.3234	4.2849	0.9138	0.5002	0.3323	3.3588
YRC22	0	0.4892	0.0063	0.0159	0	0.1626	0.0016	0.1029
Bacteroides	12.1049	13.2785	0.195	0.3126	2.1714	4.8599	0.0873	0.169
Paludibacter	0	2.0549	0.0153	0.0475	0	0.088	0.007	0.1146
Parabacteroides	0.3366	1.0498	1.7946	2.4555	0.2802	0.5917	0.7292	1.7824
Prevotella	14.5697	23.4859	23.6714	47.1385	18.7519	32.4054	10.7415	43.4432
Alistipes	0	0	0.0247	0	0	0	0	0
Chlamydia	0	0	1.195	0.0062	0	0.0185	0.0722	0
Mucispirillum	0	0	0.0093	0	0	0	0	0
Fibrobacter	0	0	0.0275	0	0	0	0	0.0328
Bacillus	0	0	0	0	0	0	0.1824	0
Rummeliibacillus	0	0	0.0214	0	0	0	2.1184	0
Staphylococcus	0	0	0	0.0059	0	0	0	0
Enterococcus	0.0415	0.0018	0.0036	0	0.0149	0.0625	0.0125	0
Lactobacillus	27.7438	5.3235	7.548	4.7266	21.8496	13.669	28.4202	7.8896
Pediococcus	0.0246	0	0.1947	0	0.0291	0	0.5799	0
Weissella	0	0	0.0929	0.0111	0	0	0.1774	0.0209
Streptococcus	1.3399	0.4043	0.047	1.9864	0.4434	0.1273	0.0401	1.8217

Turicibacter	0	0.0345	0.0842	0	0	0.0268	0	0
Mogibacterium	0.0026	0.0163	0.0076	0.0111	0.1979	0.0045	0.0046	0
Anaerococcus	0.0099	0	0	0	0	0	0	0
Peptoniphilus	0.0589	0	0	0	0.0249	0	0.0011	0
Christensenella	0.0011	0	0	0	0.03	0	0	0
02d06	0.0781	0.0018	0.0045	0.1127	0.0014	0	0.0011	0.0961
SMB53	0.0065	0	0	0.0341	0	0.0582	0	0
Dehalobacterium	0	0.014	0	0	0	0	0	0
Anaerofustis	0	0	0.0294	0.0046	0	0	0.0349	0.0101
Pseudoramibacter_Eubacterium	0	0	0	0	0	0	0	0.0119
Anaerostipes	0	0	0	0	0	0	0	0.0126
Blautia	0.0605	0.1633	4.4026	1.3418	0.0566	1.5482	6.6467	1.9729
Butyrivibrio	0	0	0.0106	0.0093	0	0	0.0059	0.0381
Clostridium	2.3579	0.1589	0.2037	2.4083	1.6163	0.2866	0.0789	3.0117
Coprococcus	0.0429	0.0106	0.2361	3.0349	0.0374	0.1588	0.3042	3.6081
Dorea	1.0008	0.0808	0.4716	1.135	1.5016	0.3193	0.7156	1.135
Lachnospira	0	0.0061	0.0811	1.8007	0	0.0046	0.5338	1.9835
Roseburia	2.9189	0.5659	0.1165	5.4741	0.1433	1.4211	0.1961	5.692
Peptococcus	0.0035	0.0448	0.0118	0	0.0255	0.0868	0.0294	0
rc4-4	0.0024	0	0	0.0227	0	0.0138	0	0.005
Peptostreptococcus	0.0826	0.0336	0	0	0	0	0	0
Anaerotruncus	0.0329	0.044	0	0	0.05	0.0214	0	0
Butyricicoccus	0.0133	0	0	0	0	0	0	0.0176
Clostridium	0	0.0075	0	0.0103	0	0	0	0.0172
Faecalibacterium	0.5302	0.0681	5.1226	4.546	0.364	1.1187	1.0098	6.7742
Oscillospira	2.0718	1.1012	0.1533	1.8155	3.1054	4.4369	0.0535	1.4136
Ruminococcus	3.4383	0.6456	2.2631	3.5648	2.8721	0.7276	0	2.6724
Acidaminococcus	0	1.3625	0.1565	0.0334	0.4257	1.0909	0.0232	0.1117
Anaerovibrio	0	5.8232	0.0165	0.3895	0.0015	0.6562	0	0.718
Dialister	0	0.0182	0	0	0.2928	0.0213	0	0

Megamonas	0	0	0.031	0	0	0	0.7642	0.0071
Megasphaera	0.0163	1.7504	0.027	0	7.6893	3.5189	0.0099	2.2641
Mitsuokella	0	0.4919	0.0079	0.0042	0.8234	1.0797	0	0.0024
Phascolarctobacterium	0.9381	1.9723	4.3555	1.7771	1.6318	4.3312	0.3324	1.5958
Selenomonas	0	0.1669	0	0	0	0.005	0	0.0071
Succiniclasticum	0.0055	1.0737	0	0	0	0.0419	0	0
Veillonella	0.4341	0.0037	0	0	0.0499	0.0127	0	0
Asteroleplasma	0	0.0067	0.0055	0.0065	0.0491	0.012	0.0117	0
Bulleidia	0.0251	2.4979	1.2736	0.6565	0.0681	0.0359	6.8724	0.5759
Catenibacterium	0.0533	0.1819	15.0011	0.0669	0.105	0.2179	14.1885	0.0951
L7A_E11	0.0052	0	0	0	0.1275	0	0.0968	0.0222
p-75-a5	0.3215	0.2393	0.0711	0.0927	11.218	2.9995	0.0898	0.0472
RFN20	0.0009	0.0037	0.0432	0	0.0245	0	0.0138	0
Sharpea	0.1254	0.2107	0.1041	0	0.5924	0.4637	0.2468	0
Fusobacterium	20.1534	8.4038	0	0	1.0235	1.8596	0.0023	0
Ochrobactrum	0	0	0.007	0	0	0	0	0
Sphingobium	0	0	0	0.0104	0	0	0.0831	0
Sutterella	0.2162	0.2552	0.0064	0.0334	0.046	0.0593	0	0
Comamonas	1.3819	0.0205	0	0	0.202	0	0	0.0082
Limnohabitans	0.0098	0	0	0	0	0	0	0
Oxalobacter	0	0	0	0	0	0.0062	0.0099	0.0092
Methyloversatilis	0	0	0	0	0	0	0.0366	0
Bilophila	0.0839	0.1369	0.0015	0	0.1858	0.1027	0.0013	0
Desulfovibrio	0.5277	1.7083	0.6371	0.1812	0.9873	1.7325	0.2032	0.0827
Campylobacter	0	0.5686	0.0164	0.0261	0	0.1115	0.031	0.0081
Flexispira	0	0	0	0	0.0156	0.0164	0	0
Helicobacter	0	0.0106	0.0018	0	0	0.0544	0.0013	0
Anaerobiospirillum	0.1849	0.5289	0	0	0.0038	0	0	0
Succinivibrio	0	2.9711	0.0207	0.0203	0.0163	3.1055	0.0097	0.5302
Enterobacter	0	0.015	0	0	0	0	0	0

Actinobacillus	0.0533	0.0014	0.003	0	0.0389	0.0361	0	0
Pasteurella	0.0142	0	0	0	0.0122	0.0202	0	0
Acinetobacter	0	0	0.0474	0.0209	0.0014	0	0.0625	0
Sphaerochaeta	0.0261	0	0	0.09	0	0	0	0
Treponema	0.002	3.6264	2.0541	3.9311	11.0669	8.4102	0.7122	0.4929
Pyramidobacter	0	3.1795	0.0186	0	0	0.4987	0	0
Synergistes	0.0298	1.2282	0.0202	0	1.4978	0.0917	0.0012	0
vadinCA02	0	0.4113	0	0	0.0243	0.4587	0	0
Akkermansia	0.2662	3.9167	0	0	0.0064	0.0274	0	0
[Eubacterium]	0.5431	0.0842	5.0469	0.3839	0.251	0.3067	4.9185	0.7616
[Prevotella]	0.3502	0	0.9436	2.8906	0.4039	3.3883	0.303	3.0088
[Ruminococcus]	1.1754	0.2206	0	1.7946	1.1142	0.265	1.4052	1.1959
